# Chlorogenic acid/chromium supplement rescues diet-induced insulin resistance and obesity in mice

**DOI:** 10.1186/s12986-015-0014-5

**Published:** 2015-05-22

**Authors:** Hilda E Ghadieh, Zachary N Smiley, Melissa W Kopfman, Mona G Najjar, Michael J Hake, Sonia M Najjar

**Affiliations:** Center for Diabetes and Endocrine Research (CeDER), College of Medicine and Life Sciences, University of Toledo, Toledo, OH 43614 USA; Department of Physiology and Pharmacology, College of Medicine and Life Sciences, University of Toledo, Toledo, OH 43614 USA; College of Medicine and Life Sciences, University of Toledo, Health Science Campus, 3000 Arlington Avenue, Mail stop 1009, Toledo, OH 43614 USA

## Abstract

Abdominal obesity is a major risk factor for insulin resistance, type 2 diabetes and cardiovascular diseases. Dietary fat induces insulin resistance in humans and rodents. The current study investigates whether a Chlorogenic acid/Chromium III supplement rescues obesity and insulin resistance caused by high-fat feeding of male C57BL/6 J mice for 7 weeks. Administering an oral daily dose of this supplement in the last 3 weeks of feeding reversed diet-induced body weight gain and insulin resistance, assessed by hyperglycemia, glucose intolerance and insulin intolerance. Indirect calorimetry analysis revealed that this effect is mediated at least partly, by increasing energy expenditure and spontaneous locomoter activity. These findings underscore the important role that chlorogenic acid and chromium play in maintaining glucose metabolism and insulin response in mice fed a high-fat diet.

## Background

The prevalence of visceral obesity has steadily increased worldwide. Currently, the majority of Americans are overweight and nearly 35 % are obese [1, 2]. Because abdominal obesity constitutes an increased risk of developing type 2 diabetes and fatty liver disease that are spreading at an epidemic rate [3], it has become imperative to develop means that could be easily introduced to our daily lives to fight efficiently the spread of this disease.

Abdominal obesity is commonly associated with peripheral insulin resistance, a hallmark of metabolic syndrome and related diseases. Peripheral insulin resistance is multifactorial and is manifested by hyperinsulinemia and random hyperglycemia in addition to glucose and insulin intolerance [4]. The cause-effect relationship between obesity and insulin resistance has been a subject of intense investigation. It has been widely accepted that increase in caloric burden promotes lipid storage in white adipose tissue (WAT), causing visceral obesity followed by insulin resistance. Diet-induced insulin resistance is mediated by several mechanisms, including the release of WAT-derived fatty acids and adipokines that blunt insulin signaling and cause ectopic lipotoxicity and a systemic pro-inflammatory state [5]. This also reduces adiponectin release from WAT, an event that contributes to the insulin resistance state caused by high-fat intake [6].

Reciprocally, several laboratories, including ours, have reported on the metabolic and cardiovascular benefits of caloric restriction and exercise in humans and rat models of metabolic syndrome [7–9]. However, abundance of food supply and the more favored sedentary life style have rendered weight loss a continuous challenge to inhabitants of the industrialized world. A large number of beverages and foods containing no-calorie or low-calorie (e.g., artificial or indigestible) sweeteners have been developed in recent years. However, these have failed to control body weight loss efficiently, owing to complex body feedback systems that may eventually stimulate food craving and excessive caloric intake [10, 11].

Polyphenols containing natural products exert an anti-diabetic effect [12]. Coffee contains the highest concentration of polyphenols among other beverages [13]. Chlorogenic acid (CGA) is a phenolic metabolite extracted from some plant species, such as *Coffea Canephora Pierre* (commonly known as green coffee beans). CGA has many biological properties (i.e. antioxidant) [14] and insulin-sensitizing activities [15]. It also reduces hyperglycemia via several mechanisms [16]. These include stimulating glucagon-like peptide 1–mediated insulin secretion [17] and activating AMP-dependent kinase to promote translocation of glucose transporter 4 to the plasma membrane for glucose uptake [18].

Yet, increased consumption of coffee alone may be counter effective in some individuals, as caffeine can cause sleep deprivation, which in turn, causes obesity and insulin resistance [19–21]. Thus, coffee was supplemented with CGA and chromium III (CrIII), a rapidly absorbed chromium form with an anti-inflammatory effect [22], to investigate whether this CGA/Cr formulation reverses the metabolic abnormalities induced by high-fat diet in C57BL/6 J (BL6) mice, an animal model that responds metabolically to dietary fat like humans [23]. We herein demonstrate that supplementing diet with daily oral gavage of CGA/Cr for 3 weeks reversed diet-induced obesity, hyperglycemia and insulin resistance, and that this is mediated at least in part, by increasing energy expenditure and inducing spontaneous physical activity.

## Methods

### Animals and feeding

C57BL/6 J (BL6) male mice (3 months of age) were fed *ad libitum* either a standard regular diet (RD) deriving 12: 66: 22 % calories from fat: carbohydrate: protein, or a high-fat diet (HF) deriving 45: 35: 20 % calories from fat: carbohydrate: protein (D12451, Research Diets, New Brunswick, NJ). Fatty acid composition in HF diet was 36: 45: 19 % saturated (SFA): monounsaturated (MUFA): polyunsaturated fatty acids (omega-6 PUFA). Mice were kept in a 12-h-dark/light cycle, and the Institutional Animal Care and Utilization Committee approved all procedures.

### Treatment

Mice were fed either HF or RD for 7 weeks. In the last 3 weeks of feeding, RD-fed mice were treated with water alone while HF-fed mice were treated with water, a CGA/Cr supplement or with CGA alone. The CGA/Cr supplement contained base caffeine [0.1 mg/mouse/day caffeine (Boresha International Inc.), 0.1 mg/mouse/day Vitamin C (Sigma-Aldrich, St. Louis, MO) and 5.13 mg/mouse/day fructose (Sigma-Aldrich)] in addition to 2.3 μg/mouse/day chromium dinicocysteinate (Cr) (Inter Health, Benecia, CA) [24] and 0.21 mg/mouse/day decaffeinated Phenol-Chlorogenic Acid comprising 3-O-caffeoylquinic acid (3-CQA), 4-CQA and 5-CQA (Naturex SA, Avignon, France) [25]. Chromium was removed from the supplement designated as CGA alone. Doses were similar to those used in humans adjusted to 40 g body weight/mouse. Mice were fasted daily for one hour before they were subjected at 1500 h to a once daily oral gavage of 0.5 ml of water, CGA or CGA/Cr, using a feeding tube. The approach of fasting and using 0.5 ml was designed to avoid aspirations.

### Intra-peritoneal insulin tolerance test

As previously described [26], mice were fasted for 7 h starting at 700 h. Human insulin Novolin (Novo Nordisk NDC 0169-1833-11) was administered intraperitoneally (IP) at 0.75 U/kg BW to awake mice. Blood glucose was measured from the tail vein by snipping the tail at 0–180 min post-insulin injection. Glucose levels were expressed as percentage to fasting levels.

### Intra-peritoneal glucose tolerance test

As previously described [26], mice were fasted overnight (from 1700 to 800 h the following day), and glucose was administered IP at 1.5 g/kg BW (50 % dextrose solution) to awake mice. Blood glucose level (mg/dl) was measured from the tail vein at 0–120 min post-glucose injection.

### Indirect calorimetry analysis

Indirect calorimetry and food intake were assessed in individually caged (CLAMS system, Columbus Instrument, Columbus, OH) awake mice at the end of the treatment period (*n* = 4/group), over a 3-day period after being acclimated for 2 days, as described [27]. Mice had access to food and water *ad libitum*. Spontaneous locomotor activity was measured with an optical beam measuring horizontal and vertical movement (XYZ-axis). Oxygen consumption (VO_2_) and CO_2_ production (VCO_2_) were sampled every 30 min. Data were represented as mean ± SEM of light (600-1800 h) and dark (1800 h-600 h) cycles.

### Statistical analysis

**Fig. 1:**
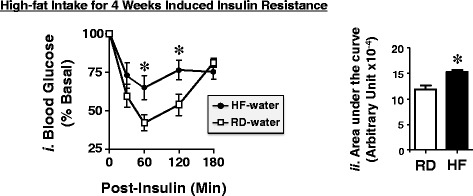
Effect of 4 weeks of High-fat Diet on Insulin Tolerance. Mice were fed either a RD (squares) or HF diet (circles) for 4 weeks before being subjected to an intra-peritoneal insulin tolerance test to assess glucose disposal at 0–180 min post-insulin injection. The area under the curve (AUC) was measured from each graph and presented in the bar graphs (panels *ii*) to the right. Values are expressed as mean ± SEM. *P <0.05 versus RD-water

Using GraphPad Prism 4 software, data were analyzed with one-way analysis of variance (ANOVA) with Bonferroni correction in all figures except for data in Fig. 1, which were analyzed by student t-test. *P* <0.05 was statistically significant.

## Results

### CGA/Chromium III and CGA supplements reversed diet-induced obesity

**Fig. 2:**
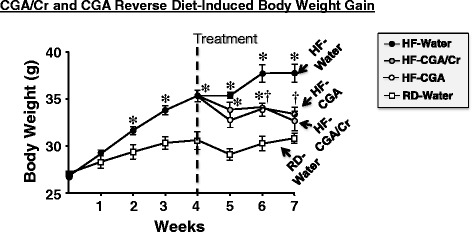
Effect of CGA/Chromium III Supplement on Body Weight. Male BL6 mice (3 months of age) were fed a regular (RD) (squares) (*n* = 7 mice) or a high-fat (HF) diet (circles) (*n* = 23 mice) for 1 month. HF-fed mice were then subjected (dashed vertical line) to gavage treatment once daily for 3 weeks with either vehicle (water) (black circles, *n* = 7 mice), CGA (hatched circles, *n* = 8 mice) or CGA/Chromium III (CGA/Cr) (gray circles, *n* = 8 mice). RD-fed mice (*n* = 7) underwent gavage with water alone. Mice continued to have full access to the same diets during the treatment period. Body weight was measured weekly over the treatment period. Values are expressed as mean ± SEM. *P <0.05 versus RD-water and ^**†**^versus HF-water

As Fig. 2 reveals, feeding mice a fat-rich diet (HF) for 4 weeks caused an increase in body weight relative to mice fed a regular diet (RD) (dark circles versus white squares). Supplementation with CGA/Cr (gray circles) and CGA (hatched circles) prevented further gain in body weight and instead, reduced it significantly within 2 weeks until it reached a level comparable to that fed a regular normal diet for 7 weeks (Fig. 2, gray and hatched circles versus white squares).

### CGA/Chromium III supplement reversed all of the metabolic abnormalities induced by high-fat diet

In addition to obesity (Fig. 2), 4 weeks of HF diet caused insulin resistance, as shown by increased intolerance to exogenous insulin relative to mice fed RD (Fig. 1,*i* black circles versus white squares, and Fig. 1,*ii* in the accompanying bar graph depicting the Area Under the Curve (AUC): 14287 ± 458 in HF-water (black bar) versus 11182 ± 691.9 arbitrary units (au) in RD-water (white bar); *P* <0.05).

**Fig. 3:**
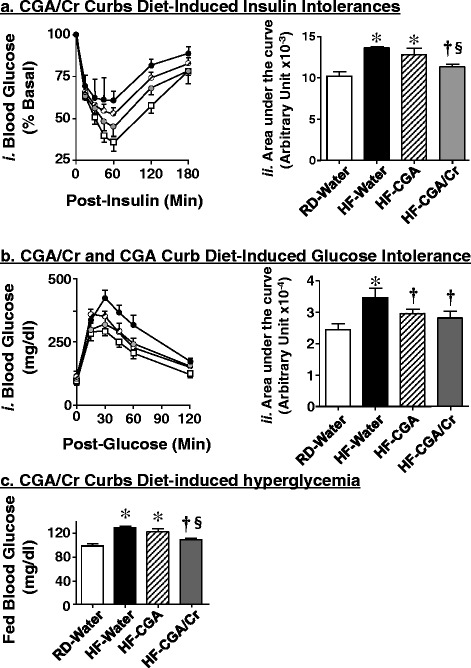
CGA/Chromium III Supplement Curbs Diet-induced Metabolic Abnormalities. Mice were fed for 4 weeks with RD (squares) or HF (circles) before receiving an oral gavage of water (black circles), CGA alone (hatched circles) and CGA/Chromium III (CGA/Cr) (gray circles), once daily as they continued to be fed the same diet for additional 3 weeks, as in the legend to Fig. 2. At the end of treatment, mice (*n* = 7-8 per each treatment per feeding group) were subjected to: **a** an intra-peritoneal insulin tolerance test to assess glucose disposal at 0–180 min post-insulin injection, **b** an intra-peritoneal glucose tolerance test to examine glucose disposal at 0–120 min post glucose injection, and (**c**) fed blood glucose levels. For (**a-b**), the area under the curve (AUC) was measured from each graph and presented in the bar graphs (panels *ii*) to the right of each graph in (**a**) and (**b**). Values are expressed as mean ± SEM. *P <0.05 versus RD-water (white bars), ^**†**^versus HF-water (black bars), and § versus HF-CGA (hatched bars)

To investigate whether CGA/Cr reverses diet-induced insulin resistance, we administered a daily oral dose of this supplement (or water alone) to HF-fed mice for 3 weeks as they continued to have free access to dietary fat. 7 weeks of HF feeding caused insulin intolerance relative to RD [Fig. 3a,*i-* black circles versus white squares and Fig. 3a,*ii*- AUC: 13611 ± 122 in HF-water (black bar) versus 10191 ± 529 au in RD-water (white bar); *P* <0.05]. CGA/Cr supplementation reduced diet-induced insulin intolerance (Fig. 3a,*i-* gray versus black circles), as demonstrated by lower AUC levels [Fig. 3a,*ii*- AUC: 11780 ± 524 in HF-CGA/Cr (gray bar) versus 13611 ± 122 au in HF-water (black bar); *P* <0.05]. Furthermore, CGA/Cr restored insulin tolerance in these HF-fed mice to a level comparable to that in mice fed a regular diet (RD-Water) [Fig. 3a,*i-* gray circles versus white squares and Fig. 3a,*ii*- AUC: 11780 ± 524 in HF-CGA/Cr (gray bar) versus 10191 ± 529 au in RD-water (white bar)]. In contrast to CGA/Cr, CGA alone did not reverse significantly insulin intolerance [Fig. 3a,*i-* hatched circles versus white squares and Fig. 3a,*ii*- AUC: 12909 ± 1126 in HF-CGA (hatched bar) versus 10191 ± 529 au in RD-water (white bar); *P* <0.05].

Prolonged HF diet caused glucose intolerance relative to RD feeding [Fig. 3b,*i-* black circles versus white squares and Fig. 3b,*ii*- AUC: 34706 ± 2906 in HF-water (black bar) versus 24434 ± 1929 au in RD-water (white bar); *P* <0.05]. CGA/Cr supplementation in the last 3 weeks of HF feeding curbed glucose intolerance [Fig. 3b,*i-* gray versus black circles and Fig. 3b,*ii*- AUC: 28137 ± 2048 in HF-CGA/Cr (gray bar) versus 34706 ± 2906 au in HF-water (black bar); *P* <0.05], and restored it to a level comparable to that in RD-fed mice [Fig. 3b,*ii*- AUC: 28137 ± 2048 in HF-CGA/Cr (gray bar) versus 24434 ± 1929 au in RD-water (white bar)]. Similarly, CGA supplementation (Fig. 3b,*i*- hatched circles) restored glucose tolerance [Fig. 3b,*ii*- AUC: 29212 ± 1113 in HF-CGA (hatched bars) versus 24434 ± 1929 au in RD-water (white bars)].

As expected, HF feeding caused fed hyperglycemia [Fig. 3c- 127 ± 2.15 in HF-water (black bar) versus 96.7 ± 4.02 mg/dl in RD-water (white bar); *P* <0.05]. CGA/Cr supplementation restored fed glucose levels [Fig. 3c- 107 ± 1.91 in HF-CGA/Cr (gray bar) versus 96.7 ± 4.02 mg/dl in RD-water (white bar)]. In contrast, CGA failed to reverse fed hyperglycemia, as assessed by persistently higher glucose levels relative to RD-water mice [118 ± 1.37 in HF-CGA (hatched bar) versus 96.7 ± 4.02 in RD-water (white bars); *P* <0.05].

### CGA/Chromium III supplement protects against diet-induced decrease in energy expenditure

**Fig. 4:**
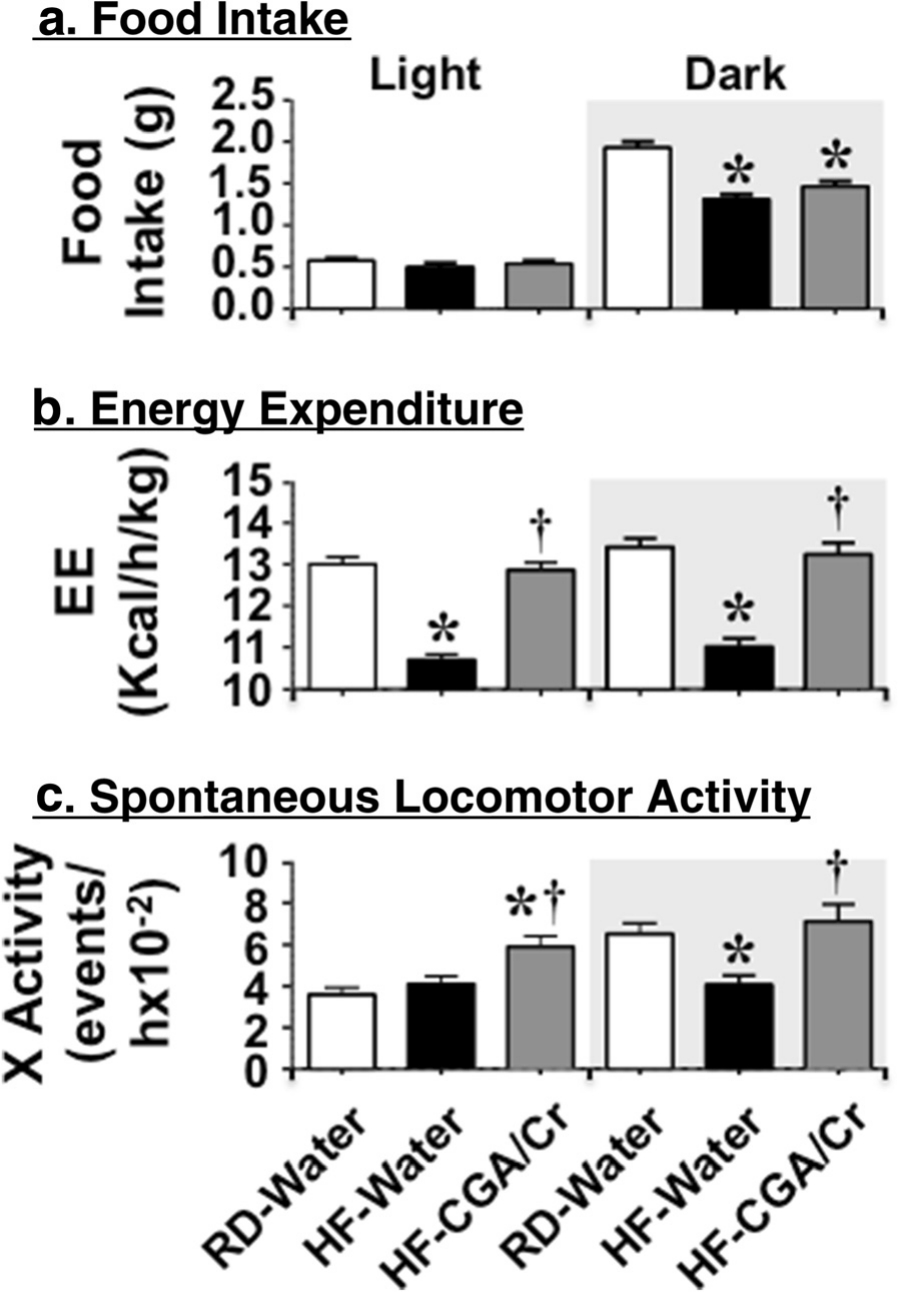
Indirect Calorimetry Analysis. At the end of the treatment period, mice were individually placed in calorimetric cages (*n* = 4/treatment group) with free access to food and water for 2 days to acclimate followed by indirect calorimetry analysis in a 24 h-period for 3 days to measure: **a** Food Intake (food g/mouse/ day), **b** Energy Expenditure (kcal/h/kg BW), and **c** Spontaneous locomotor activity (events/h) in each of the light (from 600 to 1800 h) and dark (from 1800 to 600 h-Gray shaded) cycle. Values are expressed as mean ± SEM of each time interval in the last 3 days. * *P* <0.05 versus RD-water (white bars) and ^**†**^versus HF-water (black bars)

Indirect calorimetry analysis over a 24 h-period revealed that HF feeding markedly reduced daily food intake, which is more typically intense in the dark cycle (Fig. 4a). HF feeding also reduced energy expenditure (Fig. 4b) as well as the spontaneous locomoter activity in these mice (Fig. 4c). CGA/Cr supplementation did not affect food intake (Fig. 4a), but remarkably restored energy expenditure (Fig. 4b) and the physical activity (Fig. 4c) of HF-fed mice, in particular in the dark cycle. Of note, CGA/Cr supplementation induced the physical activity of HF-fed mice even in the light cycle (Fig. 4c, gray versus black bars).

## Discussion

Obesity is a global epidemic and a major risk factor for metabolic and cardiovascular diseases. Dietary fat is a key player in the induction of insulin resistance in humans [28] and mice [29]. The current studies aimed to determine the effect of CGA/Cr supplement containing chlorogenic acid on diet-induced body weight gain and insulin resistance in BL6 mice. We herein show that CGA/Cr formulation reverses body weight gain in addition to insulin and glucose intolerance in mice fed a high-fat diet. This effect appears to be mediated, at least in part, by increasing spontaneous locomoter activity and energy expenditure.

The current study provides evidence that CGA/Cr formulation constitutes a unique alimentary combination that promotes energy expenditure and maintains physiologic insulin response even in the face of prolonged high-fat intake. This is in agreement with other reports showing the positive regulatory effect of CGA on glucose metabolism and insulin action in mice [15]. For instance, Murasi et al. [30] showed that coffee polyphenols reduced fat accumulation in BL6 mice fed a high-fat diet for 15 weeks by increasing energy metabolism and reducing lipogenesis in liver. These studies differ from ours insofar as they implicated the supplementation of the diet with purified polyphenols at the beginning of the feeding regimen, supporting a preventive effect of polyphenols on the metabolic derangement caused by long-term consumption of fat-enriched diet. However, other reports [31, 32] revealed that CGA supplementation did not attenuate obesity, glucose intolerance and insulin resistance in BL6 mice fed a high-fat diet for 12 weeks, although it improved endothelial dysfunction. The different methods of CGA administration may explain some of these disparities. In those studies, CGA was supplemented with the high-fat diet over a period of 12 weeks at 0.5 % weight/weight CGA in [31] and 1 g of CGA/kg of diet in [32]. Because mice were presumably housed in groups in those studies, the CGA dose each mouse ingested may not be as clearly defined as in our studies that implicated CGA/Cr administration by gavage to ascertain equal dose delivery to each mouse at the end of the light cycle.

More importantly, CGA was not administered alone in our studies, but rather, it was supplemented with other ingredients such CrIII. Because CrIII is a potent hypoglycemic compound that bears an anti-inflammatory activity [33], it is likely that it contributed to the effectiveness of the CGA/Cr formulation in reversing at least some of the metabolic abnormalities caused by high-fat diet. Administering CGA alone without CrIII fully restored body weight and glucose tolerance, but not insulin tolerance or fed glucose levels, which remained significantly different from RD-fed mice treated with water alone. While few studies failed to detect an insulin-sensitizing role for chromium in humans [34] and rats fed a high-fat diet for 8 weeks [35], several others highlighted its metabolic benefits. For instance, it has been shown to reduce hyperglycemia and attenuate insulin resistance in part, by inhibiting the ubiquitin-proteasome pathways mediating degradation of insulin receptor substrate 1 and 2, critical mediators of insulin signaling [22]. CrIII has also been shown to elevate circulating vitamin C and adiponectin levels, and to inhibit the inflammatory pathways in type 2 diabetic rats [24] and humans [36]. Our data underscore the complementary benefit of combining CGA to CrIII in terms of body weight loss and preserving insulin response during sustained high-fat intake, which commonly causes insulin resistance and visceral obesity. Adding Vitamin C to CGA/Cr formulation could potentiate the hypoglycemic effect of CrIII [24, 36].

Of note, CGA/Cr did not increase food intake, as would be expected of the glucose-lowering effect of CrIII, and of the addition of fructose, a low glycemic carbohydrate that does not stimulate a significant release of satiety hormones such as insulin and leptin [37, 38]. Despite prevailing controversy around the negative metabolic effect of fructose in humans and mice [39], this carbohydrate did not appear to counter-regulate the overall protective effects of the CGA/Cr formulation used in our studies. It is possible that the low dose of fructose used (~0.125 mg/kg body weight) limited its adverse metabolic effect, in particular in the presence of CGA that could conversely, overcome its negative effect [40].

As expected, indirect calorimetric analysis shows that high-fat feeding reduces food intake, energy expenditure and physical activity compared to mice fed a regular diet. CGA/Cr supplementation restored energy expenditure and the physical activity of HF-fed mice without affecting food intake. This thermogenic effect of CGA/Cr is likely to be due to CGA [30] and to caffeine [41]. The underlying molecular mechanisms are not known, but polyphenols induce CEACAM1 [42], a protein that promotes insulin sensitivity by mediating insulin clearance in liver [43]. We have observed that high-fat diet reduces CEACAM1 and that transgenic overexpression of CEACAM1 in liver prevents the negative effect of high-fat diet on energy expenditure and physical activity in mice [44]. Thus, it is reasonable to speculate that induction of hepatic CEACAM1 by polyphenols contributes to the restoration of insulin sensitivity, energy expenditure and spontaneous locomoter activity caused by CGA/Cr administration. It is also possible that administering this formulation increases spontaneous physical activity and energy expenditure (by activating sympathetic nervous outflow to adipose tissue) and this in turn, promotes insulin sensitivity [27].

In summary, the current studies provide evidence that this caffeine-based CGA/Chromium III formulation curbs the adverse effect of high-fat diet on body weight and insulin action in mice. Given that the metabolic response of BL6 mice to high-fat diet simulates that of humans, the data suggest that this CGA/Chromium III formulation could serve as a promising alimentary supplement to facilitate weight control with consumption of a fat-enriched diet. More studies are needed to investigate the underlying molecular and cellular mechanisms. Nonetheless, the current report demonstrates the important role of increased energy expenditure and physical activity by daily CGA/Chromium III consumption in preventing body weight gain and insulin resistance in response to high-fat intake.

## Conclusions

We herein identify a caffeine-based CGA/Chromium III formulation that curbs the adverse effect of high-fat diet on body weight and insulin action in mice. This is partly mediated by preserving energy expenditure and spontaneous physical activity.
